# TiO_2_ Based Nanostructures for Photocatalytic CO_2_ Conversion to Valuable Chemicals

**DOI:** 10.3390/mi10050326

**Published:** 2019-05-15

**Authors:** Abdul Razzaq, Su-Il In

**Affiliations:** 1 Department of Chemical Engineering, COMSATS University Islamabad, Lahore Campus, 1.5 KM Defence Road, Off Raiwind Road, Lahore 54000, Pakistan; abdulrazzaq@cuilahore.edu.pk; 2 Department of Energy Science & Engineering, DGIST, 333 Techno Jungang-daero, Hyeonpung-myeon, Dalseong-gun, Daegu 42988, Korea

**Keywords:** TiO_2_, 1-D nanostructures, 2-D nanostructures, hierarchical nanostructures, photocatalytic CO_2_ conversion, reactions mechanism

## Abstract

Photocatalytic conversion of CO_2_ to useful products is an alluring approach for acquiring the two-fold benefits of normalizing excess atmospheric CO_2_ levels and the production of solar chemicals/fuels. Therefore, photocatalytic materials are continuously being developed with enhanced performance in accordance with their respective domains. In recent years, nanostructured photocatalysts such as one dimensional (1-D), two dimensional (2-D) and three dimensional (3-D)/hierarchical have been a subject of great importance because of their explicit advantages over 0-D photocatalysts, including high surface areas, effective charge separation, directional charge transport, and light trapping/scattering effects. Furthermore, the strategy of doping (metals and non-metals), as well as coupling with a secondary material (noble metals, another semiconductor material, graphene, etc.), of nanostructured photocatalysts has resulted in an amplified photocatalytic performance. In the present review article, various titanium dioxide (TiO_2_)-based nanostructured photocatalysts are briefly overviewed with respect to their application in photocatalytic CO_2_ conversion to value-added chemicals. This review primarily focuses on the latest developments in TiO_2_-based nanostructures, specifically 1-D (TiO_2_ nanotubes, nanorods, nanowires, nanobelts etc.) and 2-D (TiO_2_ nanosheets, nanolayers), and the reaction conditions and analysis of key parameters and their role in the up-grading and augmentation of photocatalytic performance. Moreover, TiO_2_-based 3-D and/or hierarchical nanostructures for CO_2_ conversions are also briefly scrutinized, as they exhibit excellent performance based on the special nanostructure framework, and can be an exemplary photocatalyst architecture demonstrating an admirable performance in the near future.

## 1. Introduction

Enormous amounts of CO_2_ emissions, mainly due to industrialization and burning of fossil fuels, are considered to be a primary source of global warming [1]. Hence, fossil fuel consumption for fulfilling energy demands has led to increased atmospheric CO_2_ levels, along with depletion of respective resources. To deal with such a critical energy and environmental issue, developments in the field of renewable energy such as wind, hydel, biomass, nuclear and solar energy, are being carried out by global scientists and researchers. Among these, solar energy, in terms of its utilization in converting anthropogenic CO_2_ into value-added chemicals on a photocatalyst surface in the presence of a reducing agent, is an alluring and auspicious research area to counter environmental pollution with the possibility of matching the renewable energy infrastructure [2]. The photocatalytic CO_2_ conversion (PCC) to value-added chemicals like CO, CH_4_, C_2_H_6_, C_2_H_5_OH, C_2_H_4_, CH_3_OH, HCOOH, etc., in general mimics the concept of natural photosynthesis and is considered a subordinate of the “Artificial Photosynthesis” research domain [2,3,4,5].

Since the invention of water photocatalysis by Fujishima and Honda in 1972 [6], TiO_2_ photocatalysts have emerged as the premier and champion material with splendid properties including favorable surface area, non-toxicity, abundant availability, high stability, and cost effectiveness [7,8,9]. On the contrary, TiO_2_ nanoparticles (0-D), with a disadvantage of only UV light absorption, also exhibit drawbacks of fast electron–hole recombination, slow charge transfer, limited light trapping and difficulty in reuse/recycling [10]. Despite the development of many alternative photocatalysts to TiO_2_, TiO_2_ still remains a distinguished and premier choice because of the specific above-mentioned attributes.

In the last few years, TiO_2_-based nanostructured photocatalysts such as one-dimensional (1-D) [11,12,13], two-dimensional (2-D) [14,15,16], and three-dimensional (3-D) or hierarchical structures [17,18], have received massive attention in a variety of photocatalysis domains [19]. The distinct properties of large surface area, high aspect ratio, directional flow of photogenerated charges resulting in decreased charge recombination, light scattering, stability and improved reusability/recyclability—especially for 1-D arrays and hierarchical nanostructures—has made them valuable and worthwhile to employ in the photocatalysis research domain. Moreover, the synthesis strategies for the 1-D nanostructures are simple and facile; however, for 2-D and hierarchical nanostructured photocatalysts, fabrication is generally limited to following/adopting complicated procedures. Hence, due to their specific geometry configurations [20,21], 1-D (nanotubes, nanowires, nanorods, etc.) and 2-D (nanosheets, nanolayers, etc.) nanostructured photocatalysts provide an attractive and exemplary opportunity to overcome the limitations of 0-D nanoparticles restricting the performance of photocatalysts. Furthermore, 3-D and hierarchical nanostructures offer superb light trapping properties within specific nanostructures, resulting in a slow photon effect and improved photocatalytic performance. Despite the eminent benefits of TiO_2_ nanostructured photocatalysts, to some extent, they possess limitations with respect to the ultra violet region (a small portion of terrestrial solar spectrum) in terms of light absorption due to their wider band gap (~3.0–3.2 eV). Therefore, to overcome such limitations, similar strategies are commonly adopted as for 0-D nanoparticles, including metal and non-metal doping [18,22,23,24,25], noble metal loading [16,26,27,28,29], graphene derivative coupling [30,31,32,33], and hetero-junctioning TiO_2_ nanostructures through the coupling of low band gap materials [11,17,34,35,36,37].

In this review, the recent progress of TiO_2_-based nanostructures employed for photocatalytic CO_2_ conversion to useful/value added chemicals is briefly overviewed. The key parameters promoting the improvement of photocatalytic performance, as well as the potential benefits offered by important frameworks, i.e., 1-D, 2-D and hierarchical nanostructures, are discussed. The photocatalytic performance of the TiO_2_-based nanostructured photocatalysts, along with their reaction conditions and procedures, is summarized. In short, this review is focused on the architectural engineering of TiO_2_-based nanostructured photocatalysts, such that they offer excellent properties with enhanced photocatalytic performance.

## 2. Photocatalytic CO_2_ Conversion: Fundamentals and Mechanism

As is well established, CO_2_ in gaseous form is a thermodynamically stable molecule with a Gibbs free energy of ΔG° = −394.4 KJ∙mole^−1^ [38]. Thus, a suitable amount of energy is required to transform gaseous CO_2_ into value-added products. Equation (1) (Gibbs free energy) and Equation (2) (overpotential) demonstrate that more negative potential than E° is needed to compensate the overpotential and make the ΔG° negative enough for spontaneous conversion of CO_2_ gaseous molecule to proceed.

ΔG° = nFE°(1)

where ΔG° = Standard Gibbs free energy, n= number of electrons involved in reaction, F = Faraday constant (96485 Cmole^−1^) and E° = standard potential of the respective reaction.

η= E − E°(2)

where η = overpotential, E = required potential and E° = standard potential.

Hence, the required overpotential can be supplied to the reaction mixture in one of several ways, including as thermal energy, electrical energy, chemical energy and solar energy. Among these sources, utilization of solar energy in the presence of a photocatalyst is considered to be one of the most sustainable and cost-effective approaches. Moreover, for the reason of high stability, it is quite difficult for a photocatalyst alone to reduce CO_2_ into products; therefore, the reduction to CO_2_ proceeds with the support of reducing agents such as H_2_O, H_2_, etc. [38,39], which can regenerate the photocatalyst by readily providing the electrons in order to fill the holes. In turn, protons are released, which react with the surface-adsorbed CO_2_ and electrons to give the desired product: a relatively feasible and less energetic pathway. Such a process is commonly known as a “proton-assisted multi-electron photoreduction process”, and it is a commonly accepted mechanism for photocatalytic CO_2_ conversion. Table 1 shows the redox potentials for various CO_2_ reactions (at pH = 7.0 and E vs. NHE) with H_2_O in vapor or liquid phase [40]. It can be noted that the selectivity of the product, another important parameter, can also be manipulated by aligning the conduction band and valence band edge of the photocatalyst with respect to the redox potentials of the reactions.

As mentioned above, the conversion or reduction of CO_2_ is more feasible in the presence of reducing agents such as H_2_O, H_2_, etc. H_2_O is commonly chosen as a reducing agent because it has benefits such as being inexpensive, less dangerous than gaseous reducing agents (H_2_, H_2_S, etc.), and it only requires simple handling. Thus, a reaction mixture of gaseous CO_2_ and H_2_O (vapors or liquid) or H_2_ gas is commonly used for the photocatalytic conversion of CO_2_ into the desired products. Upon light irradiation on the photocatalyst with a CO_2(g)_/H_2_O_(g/l)_ or CO_2(g)_/H_2(g)_ mixture, the photogenerated electrons are rapidly transferred to the adsorbed CO_2_ on the photocatalyst surface, and the presence of protons (H^+^, which are provided by the reducing agents) yields the desired products. The definitive reaction mechanism for photocatalytic CO_2_ conversion is still in need of extensive research; however, based on the binding and bridging mode of CO_2_ with a photocatalyst surface, two commonly accepted pathways reported in the literature [42,43,44,45] include (i) the carbene pathway, and (ii) the formaldehyde pathway.

The possible reactions reported in the literature for the carbene pathway are presented in Equations (3)–(11). It is expected that CO_2_^●−^ radicals are formed by the quick reaction of a single electron to the surface-adsorbed CO_2_. These radicals can then react with protons/hydrogen radicals and electrons to form CO as an intermediate product, which is believed to be adsorbed onto the photocatalyst surface, further reacting with electrons and protons in a multi-step process to produce CH^●^ radical, carbene, methyl radical and finally methanol or methane as valuable products.

CO_2_ + e^−^ → CO_2_^●−^ (3)

CO_2_^●−^ + e^−^ + H^+^ → CO + OH^−^ (4)

CO + e^−^ → ^●^CO^−^ (5)

^●^CO + e^−^ + H^+^ → C + OH^−^ (6)

C + e^−^ + H^+^ → CH^●^ (7)

CH^●^ + e^−^ + H^+^ → CH_2_ (8)

CH_2_ + e^−^ + H^+^ → CH_3_^●^ (9)

CH_3_^●^ + e^−^ + H^+^ → CH_4_ (10)

CH_3_ + OH^−^ → CH_3_OH (11)

In the formaldehyde pathway, the monodentate configuration via binding of one oxygen atom to a metal (titanium) atom, or binding of materials surface oxygen atom to the carbon atom of CO_2_, favors the formation of carboxyl radical (COOH). Carboxyl radical (COOH) can react with the protons/hydrogen radicals, forming formic acid, which proceeds through the multi-step process of electron accepting and dehydration reactions to produce formaldehyde, methanol and methane. The proposed reactions are presented in Equations (12)–(21).

CO_2_ + e^−^ → CO_2_^●−^ (12)

CO_2_^●−^ + H^+^ → ^●^COOH (13)

^●^COOH + e^−^ + H^+^ → HCOOH (14)

HCOOH + e^−^ + H^+^ → H_2_OOC^●^ (15)

HC^●^OOH + e^−^ + H^+^ → HCOH + H_2_O (16)

HCOH + e^−^ → H_2_C^●^O^−^ (17)

H_2_C^●^O^−^ + H^+^ → H_2_OHC^●^ (18)

H_2_OHC^●^+ e^−^ + H^+^ → CH_3_OH (19)

CH_3_OH + e^−^ + H^+^ → ^●^CH_3_ + H_2_O (20)

^●^CH_3_ + e^−^ + H^+^→ CH_4_ (21)

## 3. One-Dimensional (1-D) Nanostructured Photocatalysts

One dimensional (1-D) TiO_2_ nanostructures, such as nanotubes, nanowires, and nanorods, have been a topic of great interest within the photocatalysis research domain, and they have a variety of applications. The key advantages offered by 1-D TiO_2_ nanostructures include large surface areas, better charge transfer, improved adsorption capacity, and extended light absorption due to the light trapping and scattering effect [46]. Until now, a moderate amount of research has been done on the development of one-dimensional (1-D) TiO_2_ photocatalysts with a key focus being on photocatalytic CO_2_ conversion to hydrocarbon fuel/useful chemicals. Several strategies encompass heterojunction formation with other visible light active photocatalysts, doping, noble metals loading and graphene coupling for attaining the key aim of improved photocatalytic CO_2_ conversion.

**Figure 1:**
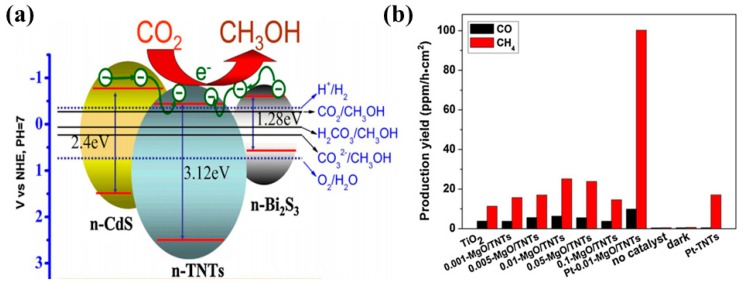
(**a**) Proposed mechanism of photocatalytic CO_2_ conversion to CH_3_OH employing CdS and Bi_2_S_3_ TNT photocatalysts (taken with permission from [47]). (**b**) Production yield of CO and CH_4_ from Pt-MgO-covered TNN films (taken with permission from [48]).

Xin et al. proposed CdS and Bi_2_S_3_ heterostructured TiO_2_ nanotubes (CdS-TNT and Bi_2_S_3_-TNT) photocatalysts prepared by a simple two-step synthesis approach [47]. TNT was first prepared using an alkaline hydrothermal method, followed by deposition of CdS and Bi_2_S_3_ in a fixed concentration using a simple precipitation approach from their respective precursors. The prepared photocatalysts were tested under visible light irradiation for photocatalytic CO_2_ conversion. The Bi_2_S_3_-TNT photocatalyst showed the maximum efficiency, yielding 224.6 µmol/L of CH_3_OH after 5 h of irradiation, as compared to CdS-TNT (159.5 µmol/L) and pure TNT (102.59 µmol/L). The increased production rate is mainly attributed to the extended light absorption of heterostructured photocatalysts and their efficient photogenerated charge separation. The conduction band edges of both CdS and Bi_2_S_3_ lie well above the conduction band edge of TiO_2_; therefore, upon light irradiation, the excited electron can easily flow to the conduction band of TiO_2_ and might react with the adsorbed CO_2_ species. A depiction of the band gap alignment and CO_2_ conversion to CH_3_OH is displayed in Figure 1a. Moreover, it was found that CdS and Bi_2_S_3_ deposition decreased the surface area and CO_2_ adsorption, in comparison to pure TNT, but did not significantly affect the photocatalytic performance.

Another effective approach is coating the 1-D photocatalyst surface with a good CO_2_ adsorbent, such as MgO-covered TiO_2_ nanotube networks (TNN) [48]. The TNN were fabricated by subjecting Ti foil to alkaline hydrothermal reaction, which resulted in well-aligned TNN. The TNN was then dipped in magnesium salt solutions of various concentrations, followed by calcination in air to finally obtain MgO-covered TNN. It was observed that MgO coverage with optimum concentration displays higher CO and CH_4_ yields when compared with bare TNN. Moreover, the deposition of platinum nanoparticles (Pt NPs) onto MgO-TNN greatly enhances the CO and CH_4_ yield. The increased performance of MgO-TNN is attributed to the chemisorption of CO_2_ molecules and their conversion to MgCO_3_ species, which are more reactive with atomic H than linear CO_2_ molecules in terms of giving the respective products. In addition, Pt NPs act as an electron trap for the efficient separation of photogenerated electron–hole pairs. The photocatalytic conversion of CO_2_ into CO and CH_4_ when employing various MgO-TNN and Pt-MgO-TNN films is displayed in Figure 1b.

**Figure 2:**
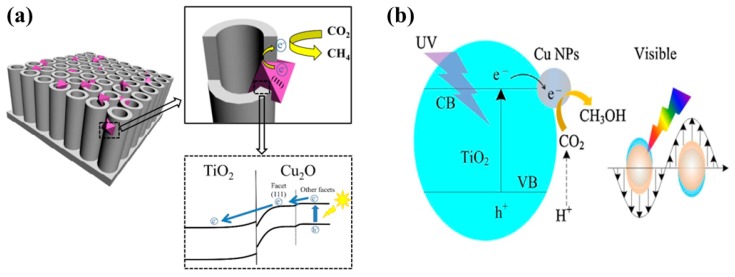
Schematic of the mechanism involved in the photocatalytic conversion of CO_2_ into: (**a**) CH_4_ employing Cu_2_O NP-incorporating TNT (taken with permission from [50]), and (**b**) CH_3_OH using Cu-modified TNF films (taken with permission from [51]).

Self-doping, such as the introduction of oxygen vacancies into the TiO_2_, generally referred to as reduced TiO_2_, is another attractive strategy for improving the photocatalytic performance. A recently published article reported the synthesis of black TiO_2_ films comprised of unique porous grid-like structures using a simple and safe hydrothermal method [49]. The elemental analysis of the synthesized TiO_2_ films displayed oxygen deficiency, indicating the presence of oxygen vacancies. When employed for photocatalytic CO_2_ conversion, the black TiO_2_ films exhibited improved CO and CH_4_ yields as compared to pure TiO_2_ film. The increased product yield was mainly attributed to the extended light absorption and the efficient charge separation resulting from the degree of defects caused by the oxygen vacancies. Another study proposed a novel heterostructure consisting of octahedral Cu_2_O nanoparticle-loaded TiO_2_ nanotube (TNT) arrays [50]. TNT arrays were prepared by a conventional electrochemical anodization method, while Cu_2_O nanoparticles were deposited from Cu salt solution using an electrodeposition approach. The Cu_2_O-TNT arrays were prepared with varied Cu_2_O deposition times. It was observed that the Cu_2_O-TNT arrays prepared with an electrodeposition time of 30 min showed the maximum CH_4_ yield under visible light irradiation. However, under simulated solar light illumination, the Cu_2_O-TNT arrays prepared with an electrodeposition time of 15 min exhibited the maximum CH_4_ yield. The key factors to which the enhanced performance was attributed include: (i) TNT arrays provide better charge transportation and light absorption, (ii) optimum loading of Cu_2_O nanoparticles leading to improved visible light performance, and (iii) well-aligned band edges of Cu_2_O and TNT arrays leading to better charge separation. The proposed mechanism involved is displayed in Figure 2a.

Another investigation reported a simple strategy for synthesizing Cu-modified TiO_2_ nanoflower films (TNF) for enhanced photocatalytic conversion of CO_2_ into CH_3_OH [51]. The synthesis methodology includes the growth of TNF on Ti foil using a hydrothermal method, followed by a microwave-assisted reduction process for Cu modification. The Cu-TNF with optimum loading of Cu (0.5 millimol concentration of Cu^2+^) exhibited enhanced CH_3_OH yield under UV-visible and UV light irradiation conditions, at a factor of 6 and 3.6 times higher than pure TNF.. The increased CH_3_OH yield was mainly attributed to good charge separation and local surface Plasmon resonance (LSPR), which were induced by Cu NPs providing hot electrons to contribute to the photocatalytic reactions. The possible mechanism involved in the photocatalytic conversion of CO_2_ into CH_3_OH is depicted in Figure 2b.

Recently, Cheng et al. reported CdS and Cu^2+^ ion deposition onto TiO_2_ nanorod (TNR) array film, and investigated its performance under visible light irradiation [52]. The TNR film was synthesized using a hydrothermal approach, while Cu^2+^ ions and CdS deposition were obtained using the cation adsorption and successive ionic layer reaction (SILAR) methods. Various samples were prepared by varying CdS deposition by SILAR cycles. The CdS-Cu^2+/^TNR film prepared with 2 SILAR cycles showed the maximum ethanol yield under visible light irradiation, thus suggesting this to be the optimum sample. Moreover, the influence of the CO_2_ flow rate and reaction temperature was also analyzed, and the optimal conditions for yielding maximum ethanol yield were found to be 4 mL/L and 80 °C.

**Figure 3:**
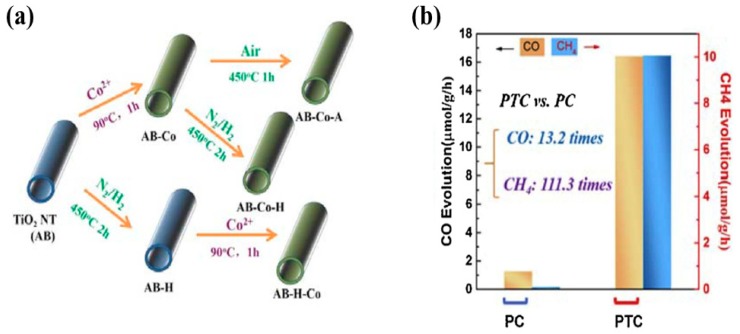
(**a**) Synthesis procedure for hydrogenated TiO_2_ nanotubes CoOx photocatalyst. (**b**) Photocatalytic CO and CH_4_ yield employing the AB-H-CoOx sample using photocatalytic and photothermal catalytic approaches (taken with permission from [53]).

Li et al. recently proposed a heterostructure of TiO_2_ nanotubes with CoO_x_, fabricated with a specially designed synthesis strategy for grafting CoO_x_ nanoparticles onto TNT and inducing defects through hydrogenation within the heterostructure by N_2_/H_2_ annealing [53]. The TNT employed in the investigation consisted of TiO_2_ (B) and anatase (A) phases. Two different samples were prepared by selecting the hydrogenation step before and after the deposition of CoO_x_ NPs, denoted AB-H-CoO_x_ and AB-CoO_x_-H, respectively. Moreover, a new concept of photothermal catalytic conversion (PTC) was employed for conversion of CO_2_ into useful products. It was observed that the TNT-CoO_x_ sample prepared using the AB-H-CoO_x_ sequence at a temperature of 393 K resulted in a greater CO and CH_4_ yield as compared to the other samples. The key parameters associated with this performance enhancement includes: (i) improved surface area, (ii) oxygen vacancies, which increase the CO_2_ adsorption on the surface sites, and (iii) upon light irradiation, the photogenerated electrons are trapped by oxygen defects, whereas the holes are rapidly transported by CoO_x_ clusters, thereby resulting in efficient charge separation. The synthesis methodology and CO_2_ conversion rates are displayed in Figure 3.

**Figure 4:**
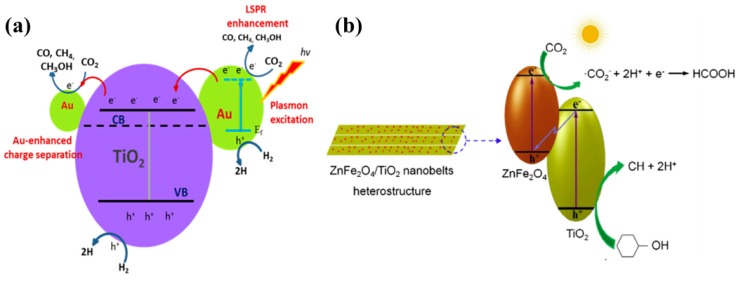
(**a**) Schematic presentation of the proposed photocatalytic conversion of CO_2_ into CO, CH_4_, CH_3_OH and other value-added chemicals employing Au-TNW (taken with permission from [54]). (**b**) Z-scheme proposed for ZnFe_2_O_4_-TNB for CO_2_ conversion to its respective products (taken with permission from [59]).

Noble metal loading, such as with silver (Ag) and gold (Au) nanoparticles, is also an effective strategy for improving the photocatalytic performance of the material. A report presented a size-controlled study of 1-D TiO_2_ nanowires (TNW) loaded with Au NPs for photocatalytic conversion CO_2_ into value-added chemicals [54]. The TNW were synthesized using an alkaline hydrothermal approach followed by deposition of Au NPs using a well-established chemical reduction method. The loading with the Au NPs was optimized by varying the concentration of the respective salts. The 0.5% Au-TNW exhibited the highest yields of CO, CH_4_ and CH_3_OH (1237, 13 and 12.6 µmol/g h, respectively) under visible light irradiation as compared to pure TNW (9, 3 and 0 µmol/g h). Moreover, the effect of TNW size was also investigated, and it was found that TNW synthesized with a reaction time of 2 h offered the best performance due to its uniform and smooth surfaces and increased surface areas. Upon loading with Au NPs, the surface area was reduced a bit, but the performance was improved due to the synergetic effect of Au NPs, with smaller-sized NPs acting as electron extractors, while larger NPs injected hot electrons into the TNW conduction band as a result of the LSPR effect. The proposed mechanism for the Au-TNW in photocatalytic CO_2_ conversion is well displayed in Figure 4a. Similarly, TNW loaded with Ag nanoparticles [55] or a combination of both Au and Ag nanoparticles [56] exhibited an analogous performance attitude under UV and visible light irradiation.. Another similar strategy involves the electrodeposition of Ag nanoparticles on the inner side of TiO_2_ nanotube (TNT) arrays [57]. The photocatalytic performance of Ag-TNT with respect to the conversion of CO_2_ was evaluated against pure TNT and Ag-TNT fabricated using a chemical bath deposition approach. The electrodeposited Ag NPs at a fixed voltage and time resulted in a uniform size distribution and good junctioning to the inner side of the TNT arrays. This heterostructure resulted in an enhanced CH_4_ yield with a small amount of CH_3_OH under light irradiation, as compared to pure TNT arrays and those of Ag-TNT arrays fabricated using conventional chemical bath deposition. The performance enhancement of the prepared photocatalyst was attributed to the light scattering inside the TNT arrays, which led to a greater degree of light absorption by Ag NPs, thus intensifying the LSPR effect. A more intensified LSPR effect leads to the injection of hot electrons into the conduction band of TNT, and thus to efficient charge separation and reaction with the adsorbed CO_2_ to yield CH_4_ or CH_3_OH. Su et al. proposed the deposition of palladium (Pd) nanoparticles onto TiO_2_ nanowires (Pd-TNW) and evaluated photocatalytic performance by CO_2_ conversion to CO and CH_4_ [58]. TNW were synthesized using a hydrothermal approach, while Pd nanoparticles were deposited using a chemical reduction method. The Pd deposition was optimized by varying the Pd salt concentration, and it was found that 0.5% Pd-TNW exhibited the maximum CO and CH_4_ yield, which was mainly attributed to the efficient electron separation of Pd from the TNW conduction band, resulting in rapid reaction with the adsorbed CO_2_.

Another research work proposed the formation of heterostructures with a visible light active photocatalyst: ZnFe_2_O_4_ nanoparticles with TiO_2_ nanobelts (TNB) [59]. The ZnFe_2_O_4_ nanoparticles were allowed to grow onto the TNB and tested for photocatalytic conversion of CO_2_ in cyclohexanol to cyclohexanone (CH) and cyclohexyl formate (CF). With the optimum concentration of ZnFe_2_O_4_ nanoparticles, i.e., 9.78%, the CH and CF yields approached their maximum; beyond this concentration, the yields decreased due to the flight shielding effect of heavy loading. The enhanced performance was attributed mainly to the efficient charge separation by a Z-scheme mechanism; Figure 4b.

**Figure 5:**
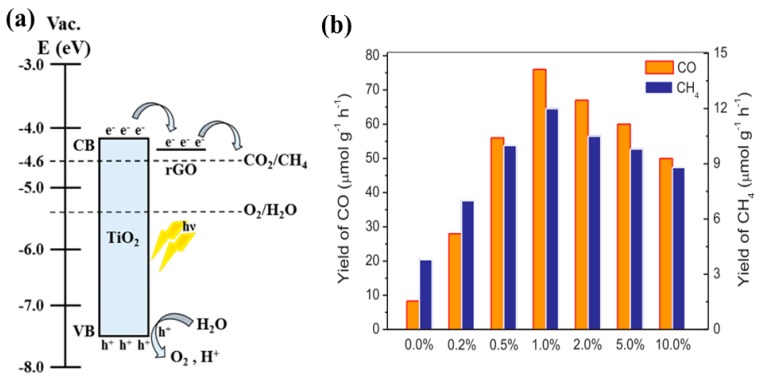
(**a**) Schematic view of photocatalytic conversion of CO_2_ into CH_4_ on rGO-TNT (taken with permission from [30]). (**b**) Production rate of CO and CH_4_ from GR-TNR with varied concentrations of graphene (taken with permission from [60]).

Another research work proposed replacing the noble metal loading with rGO sheets deposited onto TiO_2_ nanotube arrays (TNT). Interestingly, using the designed synthesis strategy, the resulting photocatalyst consisted of TNT arrays covered with rGO sheets with embedded TiO_2_ nanoparticles [30]. When employed for photocatalytic CO_2_ conversion, the resulting photocatalyst provided a CH_4_ yield that was 4.4 times higher than that achieved with pure TNT arrays, which was mainly attributed to the improved charge separation and extended light absorption. This proposed scheme for photocatalytic CO_2_ conversion is displayed in Figure 5a.

Similarly, various TiO_2_ nanostructures, i.e., nanoparticles, nanotubes and nanosheets combined with graphene, were have been synthesized and studied for the purposes of photocatalytic conversion of CO_2_ into useful products [60]. Different synthesis procedures were adopted for each nanostructure; however, TiO_2_ nanorods (TNR) were synthesized using a hydrothermal method and graphene compositing was also performed using the hydrothermal approach. The 1% graphene-TNR (GR-TNR) exhibited the highest yields of CO and CH_4_, which was mainly attributed to the increased surface area and the better interaction of graphene and TNR leading to improved charge separation under UV and visible light irradiation. The CO and CH_4_ yields from the various GR-TNR investigated are shown in Figure 5b.

**Figure 6:**
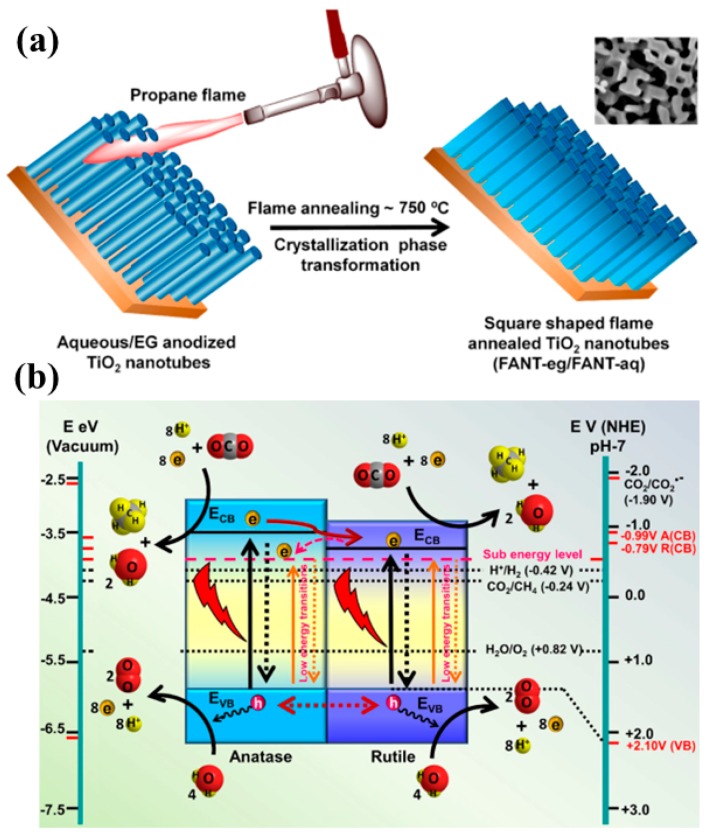
(**a**) Schematic overview of the synthesis procedure for FANT, and (**b**) the proposed mechanism for photocatalytic conversion of CO_2_ into CH_4_ employing the FANT photocatalyst (taken with permission from [61]).

Recently, Kar et al. proposed a novel strategy using flame-annealed TNT, resulting in mixed-phase (rutile and anatase) square-shaped nanotubes with defect-induced oxygen vacancies [61]. The TiO_2_ nanotubes were fabricated using a conventional electrochemical anodization method, employing a water base and an ethylene glycol base as an electrolyte. The synthesized TNT were then flame-annealed using a propane torch at a temperature of 750 °C for about 2 min. The flame-annealed TNT (FANT) using a water-based electrolyte exhibited the highest CH_4_ yield under simulated solar light and visible light irradiation of a 50 W LED lamp, and this was around 1.7 and 1.56 times higher than TNT. The improved performance of FANT (TNT prepared with a water-based electrolyte) was mainly attributed to the improved light absorption achieved by using a mixed phase of FANT, i.e., anatase and rutile, along with the well-aligned band edges of the rutile phase, which were 0.2 eV lower than in the anatase phase, resulting in efficient charge separation. The presence of Ti^3+^ states was also observed, which might have been generated due to improper oxidation of TNT; hence, extending light absorption as a result of the presence of shallow defects. Moreover, the most important aspect of the square shape of FANT is its interaction with light, resulting in a higher density of electromagnetic hotspots, and thus also contributing to improved photocatalytic performance. The synthesis procedure for FANT and the proposed mechanism involved in photocatalytic CO_2_ conversion are shown in Figure 6.

A summarized overview of the numerous TiO_2_-based 1-D nanostructures reviewed, including product type and production rate, reaction conditions, and key process parameters influencing photocatalytic performance, is presented in Table 2.

## 4. Two Dimensional (2-D) Nanostructured Photocatalysts

Fabrication of two-dimensional (2-D) nanostructured photocatalysts is another interesting and effective strategy for achieving improved photocatalytic activity. The relevant research domains have been widely investigated due to the intriguing properties they offer, which include large surface area, improved surface adsorption, improved interfacial charge transfer, and band edge alignment leading to product selectivity. With regard to photocatalytic CO_2_ conversion, a moderate amount of work has been done on the development of TiO_2_-based 2-D nanostructured photocatalysts with improved performance resulting from combinations of the factors mentioned above.

**Figure 7:**
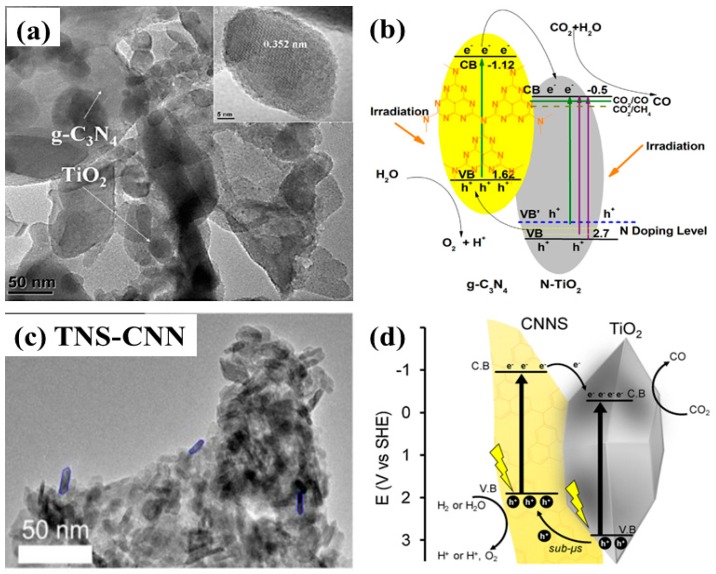
(**a**) TEM image showing the loading of N-TiO_2_ nanoparticles onto g-C_3_N_4_ nanosheets, and (**b**) proposed scheme involved in photocatalytic CO_2_ conversion (taken with permission from [62]). (**c**) TEM image showing the 2-D nanostructure of TiO_2_ nanosheets coupled with g-C_3_N_4_ nanosheets, and (**d**) schematic view of the interfacial charge transfer within TNS-CNN with the proposed photocatalytic CO_2_ conversion mechanism (taken with permission from [63]).

Loading TiO_2_ nanoparticles onto graphitic carbon nitride (g-C_3_N_4_) layered nanosheets was investigated by Zhou et al. for the purposes of photocatalytic CO_2_ conversion [62]. Various photocatalysts were prepared with varied amount of urea as a precursor of g-C_3_N_4_. Interestingly, it was observed that when the amount urea was lower than a certain limit, it acted as an N-doping source of TiO_2_, leading to the formation of N-doped TiO_2_; however, upon increasing the urea concentration, a composite of g-C_3_N_4_ and N-TiO_2_ was obtained. Such variation resulted in the selectivity of the product, yielding CH_4_ for N-TiO_2_ and CO for the g-C_3_N_4_/N-TiO_2_ composite. The product selectivity is attributed to the alignment of the band edges with respect to the redox potentials of prospective products. The g-C_3_N_4_/N-TiO_2_ with 70:30 mole ratio resulted in the maximum yield of CO only, and no CH_4_. However, the sample prepared with 50:50 mole ratio exhibited production of both CO and CH_4_. The increased photocatalytic yield of CO and CH_4_ was mainly attributed to the visible light absorption, moderate surface areas, enhanced interfacial charge transfer, and the alignment of band edges in accordance with product redox potentials. The transmission electron microscopy (TEM) image of the layered structure of N-TiO_2_ nanoparticles loaded onto g-C_3_N_4_ nanosheets is displayed in Figure 7a. The proposed mechanism for the g-C_3_N_4_/N-TiO_2_ is shown in Figure 7b.

Similarly, another recently published report proposed an in situ pyrolysis approach for the synthesis of a TiO_2_ nanosheets-g-C_3_N_4_ nanosheets nanostructure (TNS-CNN) [63]. Figure 7c shows the TEM image for the TNS-CNN, clearly displaying the fractions of TNS on the CNN. TNS-CNN has been employed for photocatalytic CO_2_ conversion using both H_2_O and H_2_ as reducing agents. It was observed that TNS-CNN provided a CO yield almost 12 times higher than pristine TNS and 37% higher than TiO_2_-P25 (with H_2_ as a reducing agent). When H_2_O was employed as a reducing agent, the CO yield decreased to one third that obtained when using H_2_ as a reducing agent. This is mainly attributed to the competition for H_2_ produced as a side reaction of water splitting, along with its reducing capability. The overall performance might be attributed to the increased surface area, the enhanced interfacial charge transfer between the nanosheets, the effectiveness of H_2_ as a reducing agent, and the visible light absorption of the CNN. A schematic representation of the CO_2_ conversion mechanism is shown in Figure 7d.

**Figure 8:**
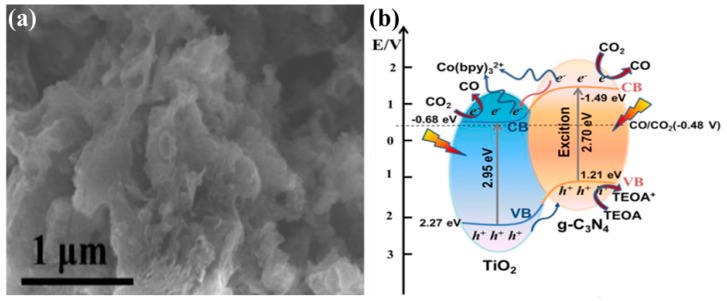
(**a**) SEM image of a representative TiO_2−x_/g-C_3_N_4_ sample displaying sheet-type g-C_3_N_4_ embedded with TiO_2−x_ nanoparticles, and (**b**) schematic of the electronic structure of TiO_2−x_/g-C_3_N_4_ with the proposed mechanism of photocatalytic CO_2_ conversion (taken with permission from [64]).

Recently, Shi et al. reported defect-rich TiO_2_ quantum dots (QDs) embedded within g-C_3_N_4_ nanosheets (TiO_2−x_/g-C_3_N_4_) for efficient photocatalytic conversion of CO_2_ into CO under solar light irradiation [64]. The fabricated TiO_2__−x_/g-C_3_N_4_ nanostructure exhibited a superior photocatalytic performance with a CO yield 5 times higher than that of pristine g-C_3_N_4_. The TiO_2−x_/g-C_3_N_4_ was synthesized by a novel and facile strategy of in situ pyrolysis of melamine with MIL-125-NH_2_ (Ti). Various samples were prepared with varying the mass ratios. The improved photocatalytic performance was mainly attributed to the improved light absorption, which extended towards the red region, the enhanced CO_2_ adsorption due to the defective TiO_2_ QDs, the efficient charge separation, as confirmed by transient photocurrent and photoluminescence spectroscopy, and the large surface area due to the nanostructured architecture of the material. Figure 8a displays the SEM image of a representative sample of sheet-type g-C_3_N_4_ embedded with 0-D TiO_2__−x_ nanoparticles. The proposed photocatalytic mechanism is depicted in Figure 8b, which provides a clear demonstration of the alignment of the band edges to the redox potentials. It can be seen that upon light irradiation TiO_2−x_/g-C_3_N_4_ generates electron–hole pairs, and the electrons from the conduction band of g-C_3_N_4_ flow towards the conduction band of TiO_2__−x_, where in the presence of Co(bpy)_3_^2+^, the co-catalyst reacts sharply with the adsorbed CO_2_ to provide CO. In contrast, the holes within the TiO_2−x_ and g-C_3_N_4_ are regenerated by the Triethanolamine (TEOA) hole scavenger.

**Figure 9:**
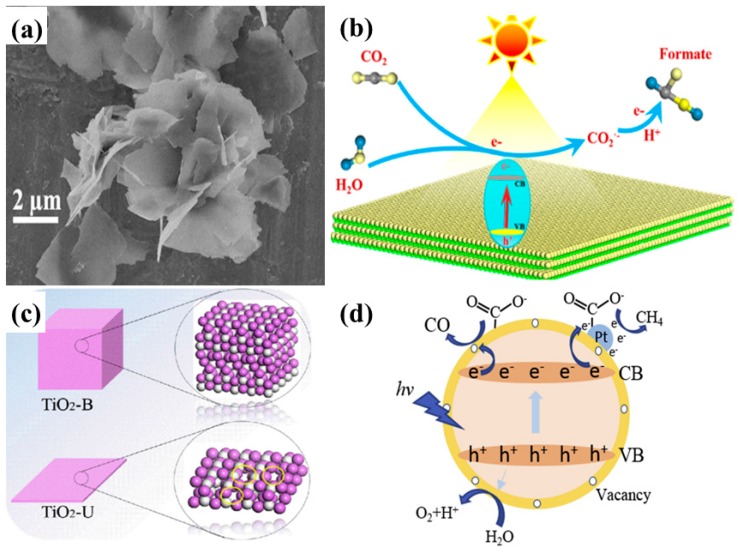
(**a**) SEM image of the TiO_2_-Octylamine hybrid nanostructure, and (**b**) schematic view of photocatalytic conversion of CO_2_ into formate product using ultrathin TiO_2_ nanosheets (taken with permission from [65]). Schematic view of (**c**) unsaturation of ultrathin nanosheets with the appearance of oxygen vacancies, and (**d**) photocatalytic CO_2_ conversion to CH_4_ with water vapors employing ultrathin TiO_2_ nanosheets with highly dispersed Pt nanoparticles (taken with permission from [66]).

Ultrathin TiO_2_ nanosheets, prepared from a TiO_2_-Octylamine lamella structure, resulted in an efficient photocatalyst for CO_2_ conversion [65]. The extravagant increase in the surface area of the TiO_2_ nanosheets led to greater light absorption and an increased number of CO_2_ adsorption active sites. It was observed that, in order to obtain ultrathin nanosheets, decreasing the bulk thickness towards atomic-scale thickness might provide the surface atoms with efficient surface active sites for photocatalytic CO_2_ conversion. Moreover, the fluorescence lifetime of the photogenerated charge within the ultrathin TiO_2_ nanosheets was observed to be higher when compared to their bulk counterparts; therefore, suggesting that the ultrathin nanosheets provide an efficient charge separation pathway along its 2-D channels. As a result of the contributions of such parameters, the formate formation from ultrathin TiO_2_ nanosheets was around 450 times higher than that of bulk TiO_2_. Figure 9a shows a SEM image of ultrathin TiO_2_ nanosheets, while Figure 9b displays a schematic view of the CO_2_ conversion mechanism.

Recently, Liu et al. reported the fabrication of TiO_2_ ultrathin nanosheets (TiO_2_-U) using a simple hydrothermal approach followed by Pt nanoparticle deposition using a photochemical deposition method [66]. The Pt-TiO_2_-U, when employed for photocatalytic CO_2_ conversion, resulted in increased CO (54.2 µmol/g h) and CH_4_ (66.4 µmol/g h) yields when compared to the pristine and reference samples. It was observed that TiO_2_-U exhibited visible light absorption, indicating the presence of oxygen vacancies, which were confirmed by X-ray photoelectron spectroscopy (XPS) and electron paramagnetic resonance (EPR) analysis. The appearance of such self-defects (oxygen vacancies or Ti^3+^ states) was attributed to the narrowing of the thickness, leading to the formation of uncoordinated surface sites, as shown in Figure 9c. Such defects with respect to Ti^3+^ promote the adsorption of CO_2_ and the uniform deposition of Pt nanoparticles. The improved photocatalytic performance was mainly attributed to the increased surface area, extended light absorption and CO_2_ adsorption sites, and enhanced electron–hole separation by Pt nanoparticles. A schematic view of the photocatalytic mechanism is shown in Figure 9d.

Surface modification of the photocatalyst is another effective approach, leading to improved surface characteristics for photocatalytic CO_2_ conversion [67]. One research work reported that the acidification of TiO_2_ nanosheets using sulfuric acid led to enhanced CH_4_ yield after 4 h of light irradiation. Acidification did not affect the sheet structure or the exposed facet of the material. However, the surface area decreased a bit due to acid molecules plugging the pores. Moreover, the acid treatment extended the light absorption of the material towards the red region; this was mainly ascribed to the generation of oxygen vacancies during the process. Such vacancy formation also enhanced the electron–hole separation under light irradiation, leading to improved photocatalytic performance.

**Figure 10:**
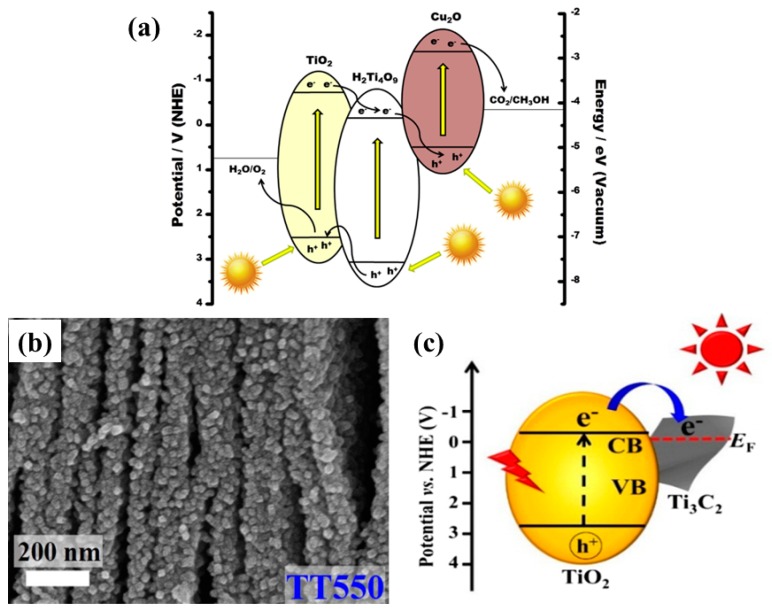
(**a**) Schematic view of the band energy diagram for Cu_2_O-loaded TiO_2_ pillared photocatalysts for conversion of CO_2_ into value-added chemicals (taken with permission from [68]). (**b**) FESEM image for TT550 sample displaying the layered structure of MXenes loaded with TiO_2_ nanoparticles, and (**c**) the proposed mechanism of photogenerated charge transfer within the TiO_2_-Ti_3_C_2_ 2-D nanostructure (taken with permission from [69]).

Lamellar structures also promote the photocatalytic activity by adsorbing photoactive species within the layers. Such layered structures with improved surface area and loading with visible active materials can lead to efficient photocatalysts, as proposed by Junior et al. [68]. The authors reported a TiO_2_ pillared K_2_Ti_4_O_9_ 2-D structure loaded with visible light-active Cu_2_O nanoparticles. The introduction of TiO_2_ pillars into layered K_2_Ti_4_O_9_ drastically increased the surface area, which was further increased after loading with Cu_2_O nanoparticles. Also, the loading with Cu_2_O nanoparticles shifted the light absorption toward the red region, along with efficient charge separation at the interface of TiO_2_, K_2_Ti_4_O_9_ and Cu_2_O. All the mentioned parameters improved the photocatalytic yield of CH_3_OH from moist CO_2_, with a yield 2 times as high as that obtained from the pristine sample. The schematic depiction of the mechanism involved is presented in Figure 10a.

Another interesting and excellent approach to developing 2-D nanostructures is the growth of photocatalytic material onto a 2-D conductive substrate. Recently, Low et al. reported a simple approach for synthesizing TiO_2_ nanoparticles coated onto a conductive Ti_3_C_2_ MXenes (TT) [69]. The TiO_2_ nanoparticles appeared as the Ti_3_C_2_ MXenes were calcined at different temperatures and tested for photocatalytic CO_2_ conversion. When employed, the photocatalytic yield of CH_4_ was found to be 3.7 times higher than that of commercial TiO_2_-P25. Figure 10b displays the SEM image of the TT sample calcined at 550 °C, which was the optimum sample. It can be seen that, upon calcination, TiO_2_ nanoparticles appear on the surface and edges of the layered Ti_3_C_2_ MXenes, resulting in a rougher surface and an improved surface area. Upon testing for photocatalytic CO_2_ conversion, the optimum sample, TT550, yielded the maximum CH_4_ production, with minor production of CH_3_OH and C_2_H_5_OH. Increasing the temperature beyond this point, the photocatalytic performance decreased due to the lower content of conductive Ti_3_C_2_ MXenes. Therefore, it can be concluded that the presence of Ti_3_C_2_ MXenes as a conductive pathway offered efficient separation of electron–hole pairs, leading to improved performance. In addition, the improved surface area contributed significantly by providing more reactive sites for CO_2_ adsorption and conversion. The proposed scheme for photocatalytic CO_2_ conversion is displayed in Figure 10c.

Another study reported Bi_2_WO_6_-TiO_2_ binanosheets (BT) as a 2-D nanostructure for photocatalytic conversion of CO_2_ into CO and CH_4_ [70]. The study mainly aimed to provide an enlightened view regarding carbonaceous intermediates or surface species in the generation of value-added chemicals. However, when tested under CO_2_ gas, it was observed that the BT sample exhibited enhanced yields of CO and CH_4_ when compared to the pristine samples. The key causes were ascribed to the improved interfacial charge transfer and the Z-scheme mechanism, which were deemed to be solely responsible for the enhanced photocatalytic performance. Table 3 provides a summarized overview of the numerous TiO_2_-based 2-D nanostructures employed for photocatalytic conversion of CO_2_ into various products, with their production rate, reaction conditions and the key process parameters promoting photocatalytic performance.

## 5. Hierarchical Nanostructures: A Dynamic and Potent Approach

In recent years, hierarchical nanostructures have received wider attention for the purpose of heterogeneous photocatalysis, as they possess admirable and exemplary properties at the micro/nanometer scale. Since the invention of 3-D/hierarchical nanostructures, intensive research has been carried out with the aim of developing competent and efficient hierarchical nanostructures for a variety of photocatalysis applications [21,72]. Based on nano-sized building blocks such as nanotubes, nanorods, nanosheets, etc., these nanostructures possess superb properties, including porous and interconnected networks, high surface areas, multi-dimensional domains for efficient charge transfer, improved light harvesting, and increased reactant adsorption, which together lead to enhanced photocatalytic performance. Therefore, these captivating aspects of hierarchical nanostructures provide a potentially dynamic opportunity to pursue the development of nanostructured photocatalysts to be employed for the photocatalytic conversion of CO_2_ into useful chemicals/fuels.

**Figure 11:**
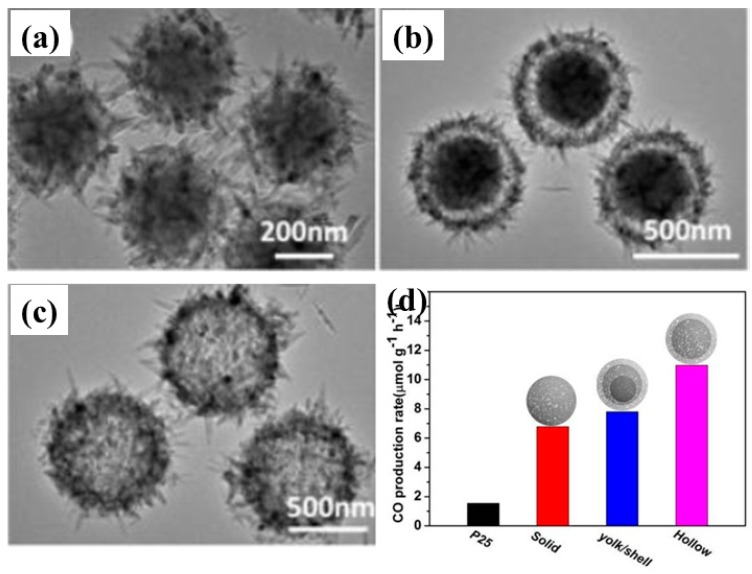
SEM images of (**a**) solid MS, (**b**) yolk/shell MS, and (**c**) hollow MS. (**d**) CO production rate for various samples via photocatalytic CO_2_ conversion (taken with permission from [73]).

Recently, Wang et al. proposed a 3-D hierarchical TiO_2_ microsphere (MS) with tunable pore and chamber size to facilitate the diffusion of the gas and its subsequent photocatalytic conversion under simulated solar light irradiation [73]. Three different types of TiO_2_ microspheres were prepared, i.e., solid MS, yolk/shell MS and hollow MS. Photocatalytic activities were tested under both UV and simulated solar light irradiation. It was observed that under UV light, solid MS exhibted the maximum CO yield (17.7 µmol/g h), which was 1.2 and 1.6 times higher than that obtained with yolk/shell MS and hollow MS, respectively. This increase in yield was attributed to the intense light absorption of solid MS. However, under simulated solar light irradiation, hollow MS showed the maximum CO yield (34 µmol/g, after 3 h of irradiation), which was 1.6 and 1.4 times higher than the yields obtained with solid and yolk/shell MS, respectively. The increased photocatalytic performance under simulated solar light was ascribed to the increased pore size of the hollow MS, leading to rapid diffusion of CO_2_ molecules towards active sites, which is a result of its unique hierarchical nanostructure. Figure 11 shows SEM images of all of the MS samples and their CO production rate under simulated solar light irradiation.

**Figure 12:**
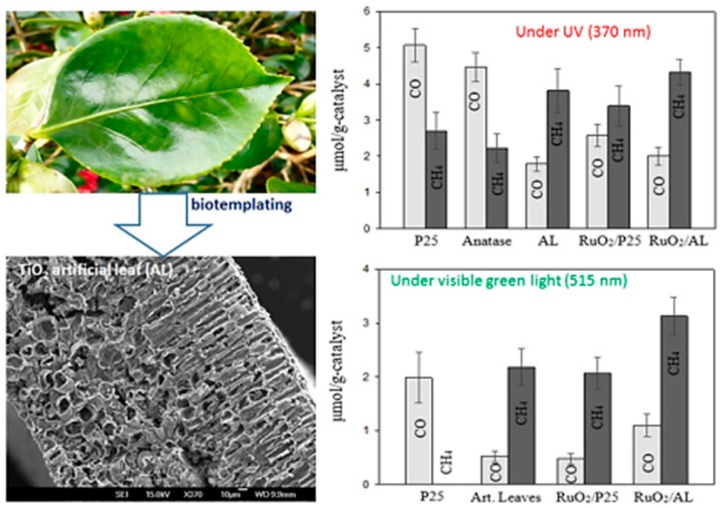
Image of *camellia* leaf, SEM image of artificial leaf displaying a porous structure, and the production rate of CO and CH_4_ via the photocatalytic conversion of CO_2_ and water vapors (taken with permission from [74]).

Similarly, a recent report presented the replication of *Camella* tree leaves for synthesis of a unique porous TiO_2_ architecture with enhanced photocatalytic conversion of CO_2_ into CH_4_ and CO [74]. The TiO_2_ artificial leaves (AL) were synthesized using a bio-template approach. The unique architecture of the AL consists of interconnected nanosheets, leading to improved porosity and a surface area greater than that of TiO_2_-P25. The photocatalytic CO_2_ conversion was tested under both UV and visible light irradiation. Under UV light irradiation, the AL yielded mostly CH_4_, whereas reference nonporous P25 yielded CO. This difference was attributed to the increased residence time and contact between reactant and catalyst within the porous network of the AL. Similar results were obtained when irradiated with visible green light; however, upon loading the Ru_2_O nanoparticles, the CO and CH_4_ yields with AL were drastically increased, which can be attributed to the efficient charge separation at the metal–semiconductor junction. Thus, the factors of porous networks with surface defects, increased surface area and efficient charge separation lead to improved photocatalytic performance in the AL hierarchical nanostructure. Figure 12 shows an image of the porous hierarchical TiO_2_ AL obtained using the bio-template approach, and the rate of photocatalytic conversion of CO_2_ into CH_4_ and CO under UV and visible light sources.

**Figure 13:**
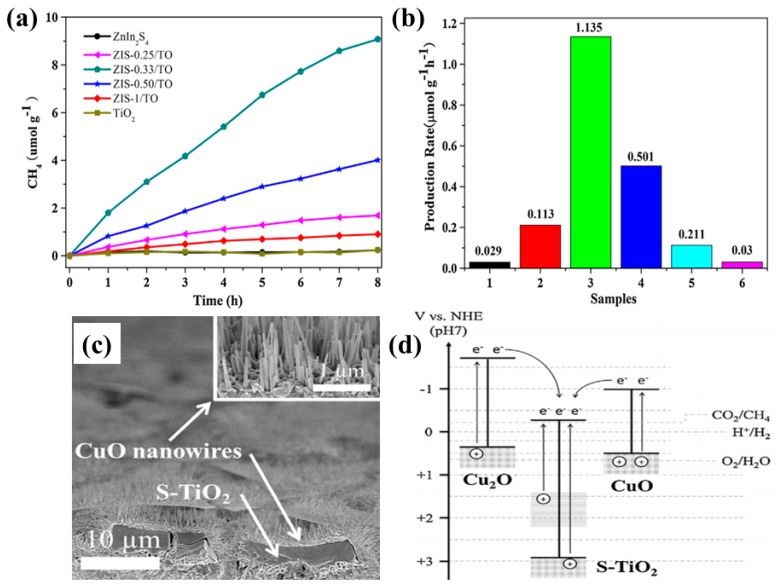
(**a**) Photocatalytic CH_4_ evolution and (**b**) production rate employing various samples with varied ratios of ZnIn_2_S_4_ to TiO_2_ (taken with permission from [75]). (**c**) SEM image of Cu_2_O/S-TiO_2_/CuO p-n-p nanostructure, and (**d**) band gap diagram with the mechanism involved in the conversion of CO_2_ to CH_4_ (taken with permission from [76]).

Another work presented the fabrication of a 3-D Z-scheme nanostructured photocatalyst consisting of ZnIn_2_S_4_ nanosheets assembled onto TiO_2_ nanobelts [75]. This hierarchical nanostructure, with an optimum ratio of ZnIn_2_S_4_ (0.33:1 mole ratio), exhibited an enhanced CH_4_ yield (1.135 µmol/g h) from the photocatalytic conversion of CO_2_ and water vapors under UV-visible light irradiation, which is around 39 times higher than the yield obtained from bare ZnIn_2_S_4_ nanosheets. The increased CH_4_ yield was attributed to the improved surface area, light absorption and effective charge separation due to the Z-scheme mechanism. Figure 13a,b shows the CH_4_ production rate from photocatalytic CO_2_ conversion.

Kim et al. developed a unique architecture composed of Cu_2_O dendrites covered with S-TiO_2_ micro-blocks and CuO nanowires, resulting in a p-n-p heterojunction formation [76]. When employed in photocatalytic CO_2_ conversion, this nanostructure yielded a high CH_4_ production rate, at 2.31 µmole/m^2^ h, which is ten times higher than with TiO_2_ nanotubes. The increased CH_4_ yield was attributed to the improved light absorption and efficient charge separation at the interfaces of the Cu_2_O dendrites, CuO nanowires and S-TiO_2_ micro-blocks. Figure 13b shows a SEM image of the nanostructured photocatalyst, while Figure 13d displays the proposed band gap diagram, depicting the charge transfer mechanisms within the nanostructure.

**Figure 14:**
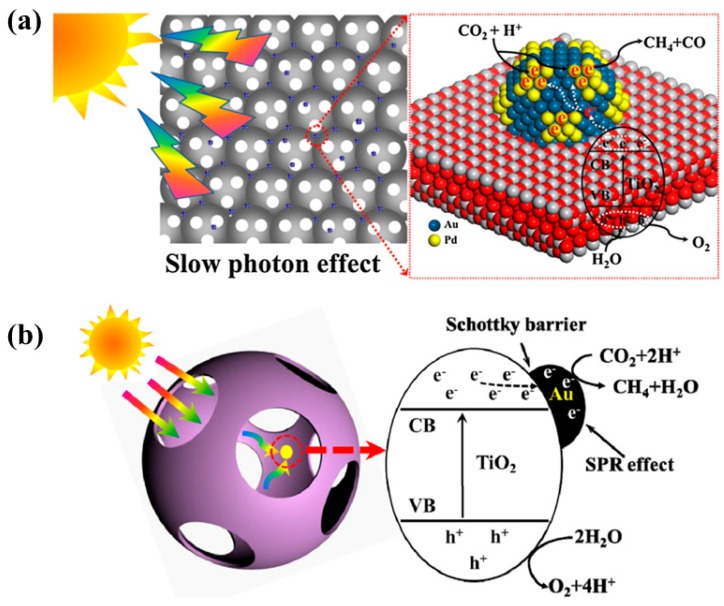
Proposed mechanism of photocatalytic CO_2_ conversion with water vapors employing (**a**) AuPd-3DOM TiO_2_ photocatalyst (taken with permission from [77]), and (**b**) Au-3DOM TiO_2_ photocatalyst (taken with permission from [78]).

The development of 3-D ordered macroporous TiO_2_ (3DOM-TiO_2_) is also an interesting strategy with the aim of improving photocatalytic CO_2_ conversion. Furthermore, the loading of noble metals like Au, Ag and Pd onto 3DOM-TiO_2_ results in an efficient photocatalyst nanostructure for improved conversion of CO_2_ into useful chemicals. As previously reported, AuPd has an optimum ratio of 3:1 weight percent. This was loaded onto 3DOM-TiO_2_, resulting in an enhanced CH_4_ yield of 18.5 µmol/g h, whereas AuPd 1:3 exhibited an increased CO yield, 14 µmol/g h, when employing CO_2_ and water vapors as reactants under UV visible light irradiation [77]. The key parameters responsible for the improved performance were deemed to be the increased light harvesting due to the slow photon effects of 3DOM-TiO_2_ and the LSPR effect induced by the noble metals, and the efficient charge extraction at the interface of the noble metals and the semiconductors. Figure 14a represents the proposed mechanism for the photocatalytic conversion of CO_2_ into CH_4_ and CO when employing 3DOM-TiO_2_ hierarchical nanostructures loaded with noble metals. Another study presented 3DOM-TiO_2_ hierarchical nanostructures loaded with Au nanoparticles as an efficient photocatalyst for the conversion of CO_2_ and water vapors into CH_4_ under visible light irradiation [78]. The sample with the optimum Au loading exhibited an increased CH_4_ yield (23.90 µmol/g h) as compared to the pristine sample (11.39 µmol/g h). This increase was attributed to the increased light harvesting due to the slow photon effect, the LSPR due to the uniformly loaded Au nanoparticles, and the effective charge separation at the interface between the Au nanoparticles and 3DOM-TiO_2_. A representation of the proposed mechanism for the photocatalytic CO_2_ conversion is depicted in Figure 14b.

## 6. Conclusions

The present review briefly reveals the influential and imperative aspects of TiO_2_-based photocatalyst nanostructures, when applied for the purposes of photocatalytic CO_2_ conversion. The recent developments in TiO_2_-based nanostructures, i.e., 1-D, 2-D, and hierarchical nanostructures, have made it clear that, based on the geometry and configuration of the nanostructured photocatalyst, improved photocatalytic performance can be ascribed to a combination of (i) large surface areas, (ii) efficient separation of photogenerated charges, (iii) directional charge transport, (iv) improved light harvesting due to light trapping/scattering, and (v) the slow photon effect. Furthermore, the fabrication of hetero-junctioned nanostructured photocatalysts, coupled with noble metals, graphene derivatives, or another semiconductor, multiplies the photocatalytic performance, thus reaping the benefits of both, i.e., the aspects of both nanostructures and hetero-junction formation. Hence, it can be established that the unique and peculiar properties of nanostructured photocatalysts are accompanied by an improvement in photocatalytic performance, and their study is a potent research domain offering excellent prospects for photocatalytic conversion of CO_2_ into value-added chemicals.

## Figures and Tables

**Table 1:** Electrochemical redox potentials (E° vs. NHE, pH = 7.0) for CO_2_ reduction into a variety of useful chemical products [4,40,41].

No.	Reactions	E° vs. NHE (V)
1	CO_2_ + 2H^+^ + 2e^−^ → HCOOH	−0.61 V
2	CO_2_ + 2H^+^ + 2e^−^ → CO + H_2_O	−0.53 V
3	CO_2_ + 4H^+^ + 4e^−^ → HCHO + H_2_O	−0.48 V
4	CO_2_ + 6H^+^ + 6e^−^ → CH_3_OH + H_2_O	−0.38 V
5	CO_2_ + 8H^+^ + 8e^−^ → CH_4_ + 2H_2_O	−0.24 V
6	2H^+^ + 2e^−^ → H_2_	−0.41 V
**Water Oxidation Reactio**		
7	2H_2_O → O_2_ + 4H^+^ + 4e^−^	+0.81V

**Table 2:** Summary of various 1-D nanostructured photocatalysts, including reaction conditions, production of value-added chemicals by photocatalytic CO_2_ conversion, and key parameters for performance improvement.

1-D Nanostructure	Light Source and Reactants	Production Rate of Value-Added Chemicals	Key Parameters for Improved Performance	Ref.
CdS/TiO_2_ nanotubes and Bi_2_S_3_/TiO_2_ nanotubes	500 W Xenon lamp CO_2_ bubbled through a solution of Sodium hydroxide and sodium nitrite	After 5 h of irradiation CH_3_OH: 102.5 µmol/L for TNT CH_3_OH: 159.5 µmol/L for CdS/TNT CH_3_OH and 224.6 µmol/L for Bi_2_S_3_/TNT	Improved surface area Light absorption enhancement Improved CO_2_ adsorption Efficient electron–hole separation	[47]
MgO amorphous layers on Pt loaded TiO_2_ nanotubes networks	300 W Hg lamp CO_2_ bubbled through water	CO: 10.4 ppm/h cm^−2^, and CH_4_: 100.2 ppm/h cm^−2^ for Pt loaded on 0.01 M MgO coated TiO_2_ Nanotube Networks	Improved CO_2_ adsorption Enhanced electron–hole separation	[48]
Black TiO_2_ films with grid-like structures	300 W Xenon lamp CO_2_ bubbled through water	CO: 115 µmol/g h, and CH_4_: 12 µmol/g h	Improved light absorption Improved charge separation due to extended charge lifetime	[49]
Cu_2_O nanoparticles modified TiO_2_ nanotube arrays	350 W Xenon lamp with and without UV cutoff filter CO_2_ bubbled through water	After 4 h of irradiation using simulated solar light CH_4_: 400 ppm/g for sample with 15 min of Cu_2_O electrodeposition After 4 h of visible light irradiation CH_4_: 8 ppm/g for sample with 30 min of Cu_2_O electrodeposition	Visible light absorption by Cu_2_O TNT providing a pathway for efficient electron–hole charge separation	[50]
Cu deposited on TiO_2_ nanoflowers films	500 W Xenon lamp with UV cutoff filter CO_2_ bubbled through water	CH_3_OH: 1.8 µmol/cm^2^ h under UV and Visible light irradiation For 0.5 Cu/TiO_2_ film	LSPR effect due to Cu nanoparticles and efficient charge transfer property	[51]
Au nanoparticles deposited on TiO_2_ nanowires	HID 35 W Car lamp CO_2_ and H_2_ gaseous mixture	CO: 1237 µmol/g h, CH_4_: 13 µmol/g h, and CH_3_OH:12.65 µmol/g h For 0.5 Au TiO_2_ NW	LSPR effect Efficient photogenerated charge extraction Improved Surface area	[54]
ZnFe_2_O_4_ nanoparticles on TiO_2_ nanobelts	250 W high pressure Hg lamp CO_2_ and cyclohexanol	After 8 h of UV illumination Cyclohexanone (CH):170.2 µmol/g and Cyclohexyl formate (CF): 178.1 µmol/g With sample containing 9.78 wt. % loading of ZnFe_2_O_4_	Improved Charge separation by a Z-scheme mechanism Enhanced Surface area	[59]
TNT arrays covered with rGO TiO_2_ nanoparticles	100 W Xenon solar simulator CO_2_ and Water vapors	CH_4_: 5.67 ppm/cm^2^ h	Efficient Charge separation Light absorption enhancement	[30]
Ag NPs TiO_2_ nanowires	35 W HID car lamp as visible light source 200 W Hg Reflector lamp as a UV light source CO_2_ and H_2_ at feed ratio of 1.0, temperature 100 °C and Pressure 1 atm.	CO: 983 µmol/g h CH_4_: 9.73 µmol/g h CH_3_OH: 13 µmol/g h From 3% Ag deposited TiO_2_ NWs	Moderate surface areas LSPR effects and efficient charge separation	[55]
Au-Ag NPs TiO_2_ nanowires	35 W HID car lamp as visible light source 200 W Hg Reflector lamp as a UV light source CO_2_ and H_2_ at feed ratio of 1.0, temperature 100 °C and Pressure 1 atm.	CO: 1813 µmol/g h CH_4_: 35 µmol/g h C_2_H_4_: 0.95 µmol/g h C_2_H_6_: 2.52 µmol/g h C_3_H_6_: 3.94 µmol/g h C_3_H_8_: 3.52 µmol/g h CH_3_OH: 18.76 µmol/g h From, 2% Ag-0.5% Au deposited TiO_2_ NWs	Improved surface area LSPR effects and efficient charge separation	[56]
Ag loaded TiO_2_ nanotube arrays (TNT)	300 W Xenon arc lamp with a 400 nm cutoff filter CO_2_ and water vapors	CH_4_: 48 mmol/h m^2^ Using the sample with electrodeposited Ag NPs at 3 V and 1 min	Schottkey Junction formation and SPR effect leading to injection of hot electrons to TiO_2_ CB	[57]
CdS QDs-Cu^2+^-TiO_2_ nanorods (NR)	300 W solar simulated Xenon lamp CO_2_ and water vapors mixture Temperature of 80 °C	C_2_H_5_OH: 109.12 µmol/g h Using sample with 2 SILAR cycles CdS QDs-0.02 M Cu^2+^ ion onto TiO_2_ NRs	Improved surface area Extended light absorption of the photocatalysts	[52]
Graphene QDs deposited TiO_2_ nanotube arrays (TNT)	100 W Xenon Solar simulator CO_2_ and water vapors	CH_4_: 1.98 ppm/h cm^2^ Employing G-TNT 3 sample prepared with 3 sec electrophoretic deposition time of GQDs	Enhanced light absorption with efficient extraction of photogenerated electron–hole pairs	[31]
Graphene TiO_2_ nanostructures including nanoparticles, nanotubes and nanosheets	300 W Xenon lamp CO_2_ and water vapors	CO: 75.8 µmol/g h CH_4_: 12.3 µmol/g h By 1% graphene TiO_2_ nanotubes	Increased surface area Increased interaction between the photogenerated electrons Improved CO_2_ adsorption	[60]
Flame annealed TiO_2_ nanotubes (FANT)	100 W Xenon solar simulator Also 50 W LED lamps employed as a visible light source CO_2_ and water vapors	CH_4_: 156 µmol/g h From FANT-aq, synthesized using water as an electrolyte medium	Visible light activity due to defect mediated performance enhancement Higher density of electromagnetic hotspots for visible light and stronger absorption of UV light	[61]
Pd-TiO_2_ nanowires (NW)	400 W Hg Lamp CO_2_ and Water vapors	After 8 h of irradiation CO: 50.4 µmol/g CH_4_:26.7 µmol/g Using 0.5% Pd-TiO_2_ NW	Pd NPs acts as an electron transfer mediator leading to improved charge transfer	[58]
TiO_2_ nanotubes (NT) coated with CoO_x_ nanoclusters	150 W UV lamp with temperature increased to 393 K CO_2_ and water vapors	After 8 h of irradiation CO: 16.403 µmol/g CH_4_: 10.051 µmol/g-s	Improved surface areas Efficient photogenerated electrons-holes separation Oxygen vacancies improved CO_2_ adsorption	[53]

**Table 3:** Summary of various 2-D nanostructured photocatalysts with reaction conditions, value-added chemicals produced as a result of photocatalytic CO_2_ conversion, and key parameters for improved performance.

2-D Nanostructure	Light Source and Reactants	Production Rate of Value-Added Chemicals	Key Parameters for Improved Performance	Ref.
g-C_3_N_4_/N-TiO_2_ nanosheets	300 W Xenon arc lamp CO_2_ bubbled through Deionized water	After 12 h of irradiation CO: 14.73 µmole/g, employing CT7 sample CO: 5.71 µmole/g CH_4_: 3.94 µmole/g, employing sample CT5	Moderate surface area Extended light absorption Efficient charge separation at the heterojunction Product selectivity due to band regulation	[62]
TiO_2_ nanosheets modified with sulfuric acid	500 W Xe Arc lamp CO_2_ and water vapors	After 4 h of irradiation CH_4_: 7.63 µmole/g	Acidification facilitates oxidation of water by Ti-OH Ti^3+^ active sites i.e., oxygen vacancies enhanced adsorption of CO_2_ Efficient charge separation	[67]
Ultrathin TiO_2_ nanosheets	300 W Hg Lamp CO_2_ bubbled through Solution of photocatalyst powder in water	Formate formation 1.9 µmole/g h 450 times higher than counterpart 9 time higher than commercially available anatase TiO_2_	Surface area increased Promoted life time of electron Efficient charge separation across the 2-D path	[65]
Cu_2_O nanoparticles loaded on TiO_2_ pillared K_2_Ti_4_O_7_ layers	Polychromatic light AM 1.5 from solar simulator CO_2_ and water vapors	After 5 h of irradiation CH_3_OH: 2.93 µmole/g 2 times more as compared to pristine sample	Increased surface area Visible light absorption Efficient charge separation	[68]
Cu modified g-C_3_N_4_ sheets with TiO_2_ nanoparticles	254 nm UV Lamp as a UV light source 500 W Xe arc lamp as a visible light source CO_2_ bubbled through the water solution containing photocatalyst sample	After 8 h of irradiation under UV light CH_3_OH: 2574 µmol/g, HCOOH: 5069 µmol/g Under Visible light CH_3_OH: 614 µmol/g, HCOOH:6709 µmol/g Optimum sample: 3 wt.% Cu and 30:70 ratio of g-C_3_N_4_ and TiO_2_	Extended light absorption and efficient charge separation by copper doping Band edges alignment reflects the selectivity for CH_3_OH and HCOOH	[71]
TiO_2_ nanoparticles on Ti_3_C_2_ nanosheets	UV LED 3 W 365 nm CO_2_ and water vapor generated in situ by reaction of NaHCO_3_ and HCl	CH_4_: 0.22 µmole/ h for 50 mg sample with small amounts of CH_3_OH and C_2_H_5_OH	Improved surface area Nanosheets providing active sites Efficient electron hole separation	[69]
Pt nanoparticles loaded ultrathin TiO_2_ nanosheets	300 W Xenon lamp CO_2_ and Water vapors	CH_4_: 66.4 µmole/h g CO: 54.2 µmole/h	26 times higher surface area Efficient electron hole separation Improved CO_2_ adsorption due to defective surface	[66]
2-D g-C_3_N_4_ with 0-D TiO_2−x_ nanoparticles	300 W Xenon lamp CO_2_ bubbled through solution containing 5 mg photocatalyst dispersed in 5 mL of solution of MeCN/TEOA with cocatalyst of Co(bpy)_3_^2+^	After 5 h of irradiation CO: 388.9 μmol/g 5 times higher than pristine g-C_3_N_4_	Promoted charge transfer due to electron channel formed between g-C_3_N_4_ and TiO_2_	[64]
